# Effect of physical exercise on social adaptability of college students: Chain intermediary effect of social-emotional competency and self-esteem

**DOI:** 10.3389/fpsyg.2023.1120925

**Published:** 2023-03-22

**Authors:** Yanying Liu, Qingkun Feng, Yao Tong, Kelei Guo

**Affiliations:** School of Physical Education and Health, Zhaoqing University, Zhaoqing, China

**Keywords:** physical exercise, social adaptability, social-emotional competency, self-esteem, college students

## Abstract

**Objective:**

To explore the relationship between physical exercise and college students’ social adaptability, as well as the mediating role of social-emotional competency and self-esteem.

**Methods:**

One thousand two hundred thirty college students were investigated by physical exercise questionnaire, social-emotional competency scale, self-esteem scale, and social adaptability scale. Data were analyzed by Pearson correlation analysis, structural equation model test and deviation-corrected percentile Bootstrap method.

**Results:**

(1) Physical exercise was positively correlated with social adaptability (*r* = 0.397, *p* < 0.01), and the direct path of physical exercise on social adaptability was significant (*β* = 0.397, *t* = 15.174, *p* < 0.01). (2) Physical exercise positively predicted social-emotional competency (*β* = 0.399, *t* = 15.235, *p* < 0.01) and self-esteem (*β* = 0.305, *t* = 10.570, *p* < 0.01). Social-emotional competency positively predicted self-esteem (*β* = 0.130, *t* = 4.507, *p* < 0.01) and social adaptability (*β* = 0.169, *t* = 6.104, *p* < 0.01). Self-esteem positively predicted social adaptability (*β* = 0.189, *t* = 6.957, *p* < 0.01). (3) Social-emotional competency and self-esteem play a significant mediating role between physical exercise and social adaptability. The mediating effect includes three paths: physical exercise→social-emotional competency→social adaptability (the mediating effect value: 0.068); physical exercise→self-esteem→social adaptability (the mediating effect value: 0.059). Physical exercise→social-emotional competency→self-esteem→social adaptability (the mediating effect value: 0.010).

**Conclusion:**

Physical exercise can not only directly affect social adaptability of college students, but also indirectly affect social adaptability through the independent intermediary role of social-emotional competency and self-esteem. Furthermore, physical exercise also affect social adaptability through the chain mediation of social-emotional competency and self-esteem.

## Introduction

Social adaptability refers to the adaptive ability of people to make psychological, physiological, and behavioral changes to better survive in society and achieve a harmonious state with society. The subject can adjust their behavior to adapt to interpersonal communication, including social ability, coping ability, interpersonal relationship ability, etc. (Chung et al., 2011). Social adaptability is the core of modern moral education and quality education and an essential goal for the healthy development of college students (Yang, 2002; Chi et al., 2016). The Chinese Ministry of Education puts forward the guiding ideology of “people-oriented, health first” in August 2002, that is, sports participation, sports skills, physical health, mental health, and social adaptation. Social adaptation is the essential overarching goal. Social adaptability is an indispensable ability for college students. It is also an important responsibility of colleges to cultivate college students’ social adaptability. High social adaptability can help college students relieve pressure, regulate negative emotions, control aggressive behavior, and promote their mental health (Ji and Zheng, 2021). However, college students with poor social adaptability often find it difficult to adapt to the school environment, easy to produce negative emotions such as inferiority, weariness, and other psychological barriers, which affect their physical and mental health (Li and Li, 2015). Therefore, exploring the influencing factors of college students’ social adaptability is conducive to promoting their positive adaptation to the environment, overcoming difficulties, and improving the mental health status of college students.

### Physical exercise and social adaptability

The physical and mental health of college students has always been a hot topic in the sports field. Researchers have generally recognized that physical exercise is an effective intervention means for college students to develop positive psychological traits and promote physical and mental development over the years. Generally speaking, college students who engage in physical exercise actively are better at communicating with others, sharing fun, establishing friendly interpersonal networks, and developing interpersonal relationships. College students who often participate in physical exercise have strong interpersonal management, and emotional regulation abilities, as well as healthy psychology (Vankim and Nelson, 2013). Therefore, they are also more socially adaptable (Ji and Zheng, 2021). Conversely, negative behavior, such as lower exercise dependence often leads to negative self-presentation, susceptibility to loneliness and social exclusion, which blocks the development of social adaptability (Liu et al., 2021). It is found that the social adaptability of college students is related to the number of times, the duration and the intensity of sports activities (Chen and Sto, 2021). The more students participate in sports activities, the better their social adaptability will be (Chen and Sto, 2021).

Although studies have explored the impact of physical exercise on college students’ social adaptability (Chen and Sto, 2021; Ji and Zheng, 2021), the research on the internal mechanism and mediating role of physical activity and social adaptability has not been fully revealed. By studying the influence of physical exercise on college students’ social adaptability and the mediating role of social-emotional competency and self-esteem between physical exercise and social adaptability, this study can not only further enrich the research scope at the theoretical level, but also provide empirical evidence for improving college students’ social adaptability at the practical level. Therefore, hypothesis 1 is proposed in this study: physical exercise can positively predict college students’ social adaptability.

### Mediating effect of social-emotional competency

Social-emotional competency refers to the ability of an individual to acquire and effectively apply knowledge, attitudes and skills to understand and manage emotions, set and achieve positive goals, express empathy for the feelings of others, establish and maintain positive relationships with peers, and make responsible decisions (Wang et al., 2019). Physical exercise has an important effect on social-emotional competency (Goh et al., 2022). Social-emotional competency mainly includes social competency and emotional competency (Choi and Gwag, 2017). Studies have shown that physical exercise has a positive effect on social competency and emotional control skills (Jiang et al., 2018; Liu J. et al., 2022). Lack of social-emotional competency is the root cause of various psychological and behavioral problems, which seriously hinder students’ academic progress and the growth of body and mind (Rautakoski et al., 2021). Improving social-emotional competency can significantly improve students’ academic performance, establish positive peer relationship, enhance emotional health and communication skills (Durlak et al., 2011), and improve social adaptability. Research has shown that social competency and emotional understanding ability can predict social adaptability (Yu, 2018). A study by Huang et al. (2021) revealed that social-emotional competency can significantly predict the social adaptability of middle school students. Therefore, social-emotional competency may be an important factor affecting social adaptability. College students who regularly participate in physical exercise have more robust social competency and emotional regulation ability, better mental health, and better social adaptability (Xiao, 2007; Yin et al., 2015). In conclusion, physical exercise may affect college students’ social adaptability through social-emotional competency. Accordingly, hypothesis 2 is proposed in this study: social-emotional competency is the mediating variable between physical exercise and the social adaptability of college students.

### Mediating effect of self-esteem

Self-esteem refers to the individual’s evaluation or emotional response to their physical characteristics, personality, social identity, and behavior. It is the psychological component of the personality self-regulation structure (Pearlin, 1989). A deeper understanding of the concept of self-esteem includes self-value, self-acceptance, self-efficacy, attitude toward oneself, and self-respect (Doré, 2017).Self-esteem can be used to measure a person’s mental health. The study confirmed that self-esteem is one of the leading contributors to anxiety for college students. Cultivating self-esteem of students is important for alleviating their anxiety issues and improving their mental health at college (Liu X. et al., 2022). Studies have found that physical exercise significantly impacts self-esteem (Shang et al., 2021; Chen et al., 2022). Physical exercise can not only enhance the physical fitness of individuals but also improve the level of self-esteem and self-concept, thus affecting the mental health of individuals (Sonstroem, 1984). The active participation of physical exercise can improve body self-evaluation, increase the level of individual self-esteem, and improve subjective well-being of college students (Shang et al., 2021).

There is a significant correlation between self-esteem and social adaptability (Zhang, 2013). As an interpersonal monitor, self-esteem can regulate interpersonal relations and make them more harmonious (Bale and Archer, 2013). Individuals with high self-esteem may also be more likely to identify and use different personal and contextual coping resources (e.g., seek and receive more social support), which may, in turn, facilitate positive coping behavior and adjustment (Boden et al., 2008), so the more socially resilient they are. It is worth pointing out that, as a kind of challenging social practice, physical exercise is often accompanied by difficulty overcoming, task challenges, self-improvement and goal realization. Therefore, individuals may constantly experience learning, coping and self-presentation during exercise practice process so as to improve self-esteem, self-concept level as well as social adaptability (Biddle and Asare, 2011; Lubans et al., 2016). It can be inferred that physical exercise can affect college students’ social adaptability through self-esteem. Therefore, hypothesis 3 is proposed in this study: self-esteem is the mediating variable between physical exercise and college students’ social adaptability.

### Chain mediating effect of social-emotional competency and self-esteem

Sociometer theory proposes that self-esteem is an adaptation that evolved to monitor and regulate interpersonal relationships (Leary and Baumeister, 2000; Mann and Blumberg, 2022). Low self-esteem is associated with social phobia (Perczel-Forintos and Kresznerits, 2017). Social competency plays a crucial role in the formation of self-esteem (Mayordomo et al., 2020). Emotional competency is also closely related to self-esteem (Barrera et al., 2019). Therefore, there is a close relationship between social-emotional competency and self-esteem (Barrera et al., 2019). It is found that the cultivation of social-emotional competency can improve individual self-esteem (Heiman and Olenik-Shemesh, 2020). In other words, social-emotional competency affects self-esteem. Therefore, hypothesis 4 is proposed in this study: social-emotional competency and self-esteem play a chain mediating role between physical exercise and social adaptability.

### Hypotheses of this study

**Figure 1:**
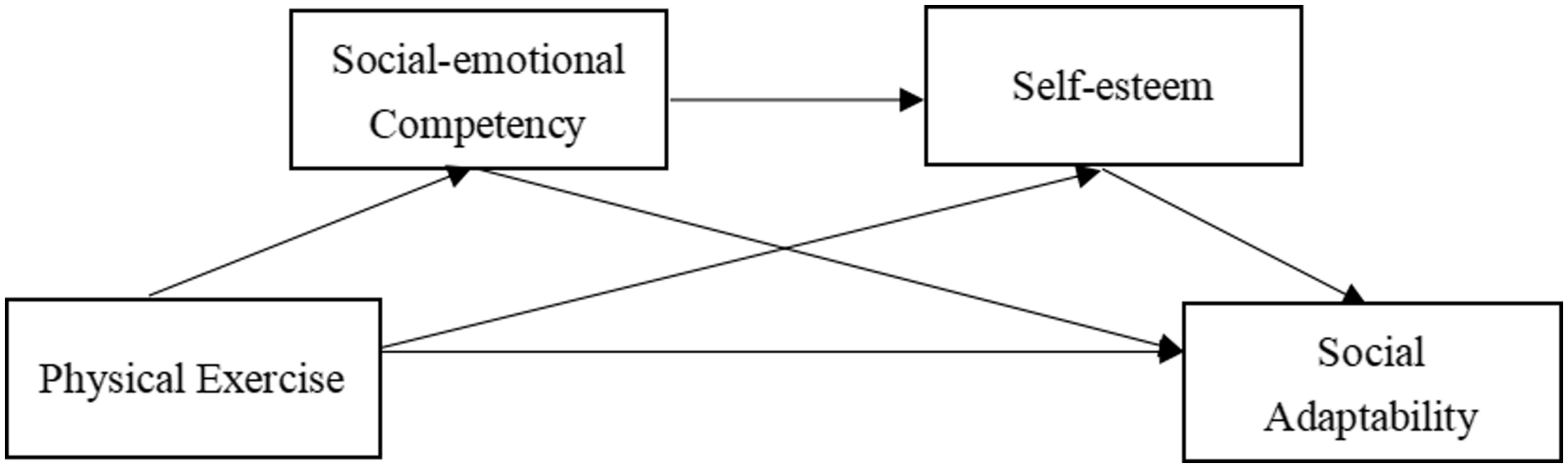
The hypothetical structure model.

To sum up, in order to investigate the internal mechanism between physical exercise and social adaptability, this study plans to build a chain mediating model (Figure 1). According to the hypothetical structure model, we will verify the following aspects: (1) Physical exercise can positively predict college students’ social adaptability; (2) Social-emotional competency plays an independent intermediary role between physical exercise and social adaptability of college students; (3) Self-esteem plays an independent intermediary role between physical exercise and social adaptability of college students; (4) Social-emotional competency and self-esteem play a chain intermediary role between physical exercise and social adaptability. In conclusion, based on verifying the above hypotheses, this study aims to explore further rrelationship between physical exercise, social-emotional competency, self-esteem, and social adaptability, to reveal the chain mediating role of social-emotional competency and self-esteem between physical exercise and social adaptability of college students, and to provide a theoretical basis for improvement of the college students’ social adaptability.

## Materials and methods

### Participants and procedure

In this study, a convenience sampling method was adopted to collect data from the students of Zhaoqing University. Pre-survey training was conducted for investigators. After the consent of the school leaders, teachers, and the subjects themselves, the group test was adopted, and the principles of voluntary filling, data confidentiality, and anonymous filling were emphasized to complete the questionnaire. A total of 1,317 questionnaires were sent out, and 1,230 valid questionnaires were collected after excluding invalid questionnaires caused by the regular responses and missing data, with an effective recovery rate of 93.40% (342 in junior one, 329 in junior two, 296 in junior three, and 263 in junior four). Among the participants, 632 were male (51.4%), and 598 were female (48.6%). The average age of the subjects was 20.39 ± 1.43 years old. This study was approved by the Research Ethics Committee of Zhaoqing University. All subjects were informed of the research purpose and signed informed consent.

### Physical exercise questionnaire

College students’ physical exercise questionnaire revised by Wu (2016) was adopted, which was adapted from the physical exercise commitment Intention Scale compiled by Chen et al. (2006). The questionnaire consists of 8 questions, including two dimensions of physical exercise commitment and persistence. The scale adopts the Likert 5 points scoring method, and each item is scored from 1 (strongly disagree) to 5 (strongly agree). The higher total score indicates a higher level of physical activity. It is proven that the scale has good reliability and validity in Chinese college students (Jiang et al., 2018). In this study, Cronbach’s α coefficient of this scale is 0.91. The fitting index of confirmatory factor analysis of the scale: *χ*^2^/df = 3.941, GFI = 0.986, AGFI = 0.973, IFI = 0.991, TLI = 0.987, CFI = 0.991, RMSEA = 0.049, indicating that the scale has good reliability and validity.

### Social-emotional competency scale

The social-emotional competency scale of this study adopted the Delaware Social Emotional Competency Scale, revised by Zhu (2016). The scale consists of 12 items, including four dimensions: social perception, self-management, peer relationship, and responsible decision-making. Likert 4-point scoring is adopted, from “1 = not like me at all” to “4 = very like me,” and “I blame others when I am in trouble” is the reverse scoring question. The higher the score is, the higher the social-emotional competency. The scale shows high reliability and validity in Chinese college students (Chu et al., 2021). In this study, Cronbach’s α coefficient of this scale is 0.90. The fitting index of confirmatory factor analysis of the scale: *χ*^2^/df = 3.240, GFI = 0.979, AGFI = 0.966, IFI = 0.986, TLI = 0.980, CFI = 0.986, RMSEA = 0.043, indicating that the scale has good reliability and validity.

### Self-esteem scale

The self-esteem scale modified by Rosenberg (1965) and Wang et al. (1999) was adopted. The scale consists of 10 questions in one dimension. Likert 4-point scoring is adopted, from “1 = completely inconsistent” to “4 = very consistent.” There are 6 forward-scoring questions and 4 reverse-scoring questions. A higher scale score indicates a higher level of self-esteem. The scale shows high reliability and validity in Chinese college students (Wan and An, 2022). In this study, Cronbach’s α coefficient of this scale is 0.85. The fitting index of confirmatory factor analysis of the scale: χ^2^/df = 4.921, GFI = 0.975, AGFI = 0.958, IFI = 0.978, TLI = 0.970, CFI = 0.978, RMSEA = 0.056, indicating that the scale has good reliability and validity.

### Social adaptability scale

The scale of social adaptability developed by Zheng (1999) was adopted. The scale consists of 20 items, including 5 dimensions: dealing with peer relationship skills, self-management skills, learning skills, obedience skills, and willingness to express skills. It aims to evaluate the social adaptability of college students from both positive and negative aspects. Likert 3-point scoring is adopted, “1 = yes” and “3 = no.” The higher the scale score is, the better the social adaptability. In this study, Cronbach’s α coefficient of this scale is 0.85.

### Statistical analysis

Statistical software Spss26.0 and Amos26.0 were used to analyze the data obtained. After the questionnaires were collected, confirmatory factor analysis was performed for all questionnaires using Amos26.0. Harman single-factor method was used to test the standard method deviation of the data. Spss26.0 was used for Pearson correlation analysis to examine the relationship among physical exercise, social-emotional competency, self-esteem, and social adaptability. Spss26.0 plug-in process was applied to examine the relationship model and mediating effect of college students’ physical exercise, social-emotional competency, self-esteem, and social adaptability. Normally distributed continuous variables are expressed as mean (M) ± standard deviation (SD). According to the references, *χ^2^/*df is less than 5, GFI, AGFI, IFI, TLI, and CFI are more significant than 0.8, and RMSEA is less than 0.08, which is acceptable. In this study, the significance level was set as *p* < 0.05.

## Results

### Common method deviation test

To ensure the accuracy of the statistical analysis results, the most commonly used Harman single-factor method was adopted in this study. A common method deviation test was carried out for the data in this paper, that is, exploratory factor analysis was done for all items of the scale together, and principal component analysis was adopted to extract components with an eigenvalue greater than 1. The test results showed that a total of 12 common factors with eigenvalues greater than 1 were extracted. The explanatory variance of the first factor without rotation was 20.737%, which was less than the critical value of 40% (Podsakoff et al., 2003). There was no single common factor to explain most of the variation. Therefore, there is no serious common method bias problem in this study.

### Descriptive statistics and correlation analysis

As shown in Table 1, the correlation coefficients of physical exercise, social-emotional competency, self-esteem, and social adaptability are all statistically significant. Correlation analysis shows that physical exercise is significantly positively correlated with social-emotional competency, self-esteem, and social adaptability. Social-emotional competency is significantly positively correlated with self-esteem and social adaptability. Additionally, there is a significant positive correlation between self-esteem and social adaptability. The correlation between variables supports the testing of subsequent hypotheses.

**Table 1:** Means, standard deviations, and correlations among variables.

Variable	*M*	SD	1	2	3	4
1. Physical exercise	22.50	6.493	1			
2. Social-emotional competency	29.60	7.499	0.399**	1		
3. Self-esteem	27.01	7.374	0.357**	0.252**	1	
4. Social adaptability	41.52	7.623	0.397**	0.321**	0.325**	1

*N* = 1,230. **p < 0.01; all tests were two-tailed.

### Examination of the mediating effects of social-emotional competency and self-esteem

According to Wen and Ye (2014)‘s suggestions on the intermediary effect test, regression analysis is carried out on the chain intermediary effect model. The process v4.1 macro program model 6 developed by Hayes (2012) was used to test the mediating effect. With social-emotional competency and self-esteem as the mediating variables, physical exercise as the independent variable, and social adaptability as the dependent variable, the stepwise regression method was used to test the mediating effect. The analysis results are shown in Table 2.

**Table 2:** Analysis of regression relationship of variables.

	Social adaptability		Social-emotional competency		Self-esteem		Social adaptability	
	*β*	*t*	*β*	*t*	*β*	*t*	*β*	*t*
Physical exercise	0.397	15.174***	0.399	15.235***	0.305	10.570***	0.262	9.133***
Social-emotional competency					0.130	4.507***	0.169	6.104***
Self-esteem							0.189	6.957***
*R*	0.397		0.399		0.376		0.469	
*R* ^2^	0.158		0.159		0.141		0.220	
*F*	230.236***		232.090***		101.076***		115.448***	

***p < 0.001.

As shown in Table 2, physical exercise can significantly and positively predict the social adaptability among college students (*β* = 0.397, *p* < 0.01). Therefore, hypothesis 1 is verified. After incorporating social-emotional competency into the regression equation, physical exercise significantly positively predicts social-emotional competency (*β* = 0.399, *p* < 0.01), and social-emotional competency significantly positively predicts social adaptability (*β* = 0.169, *p* < 0.01). Hypothesis 2 is verified. After incorporating self-esteem into the regression equation, physical exercise significantly positively predicts self-esteem (*β* = 0.305, *p* < 0.01), and self-esteem significantly positively predicts social adaptability (*β* = 0.189, *p* < 0.01). Hypothesis 3 is verified. After integrating social-emotional competency and self-esteem into the regression equation, social-emotional competency significantly positively predicts self-esteem (*β* = 0.130, *p* < 0.01), indicating the existence of chain mediation between social-emotional competency and self-esteem. Hypothesis 4 is verified. It can be concluded that social-emotional competency and self-esteem play a chain mediating role between physical exercise and the social adaptability of college students.

**Figure 2:**
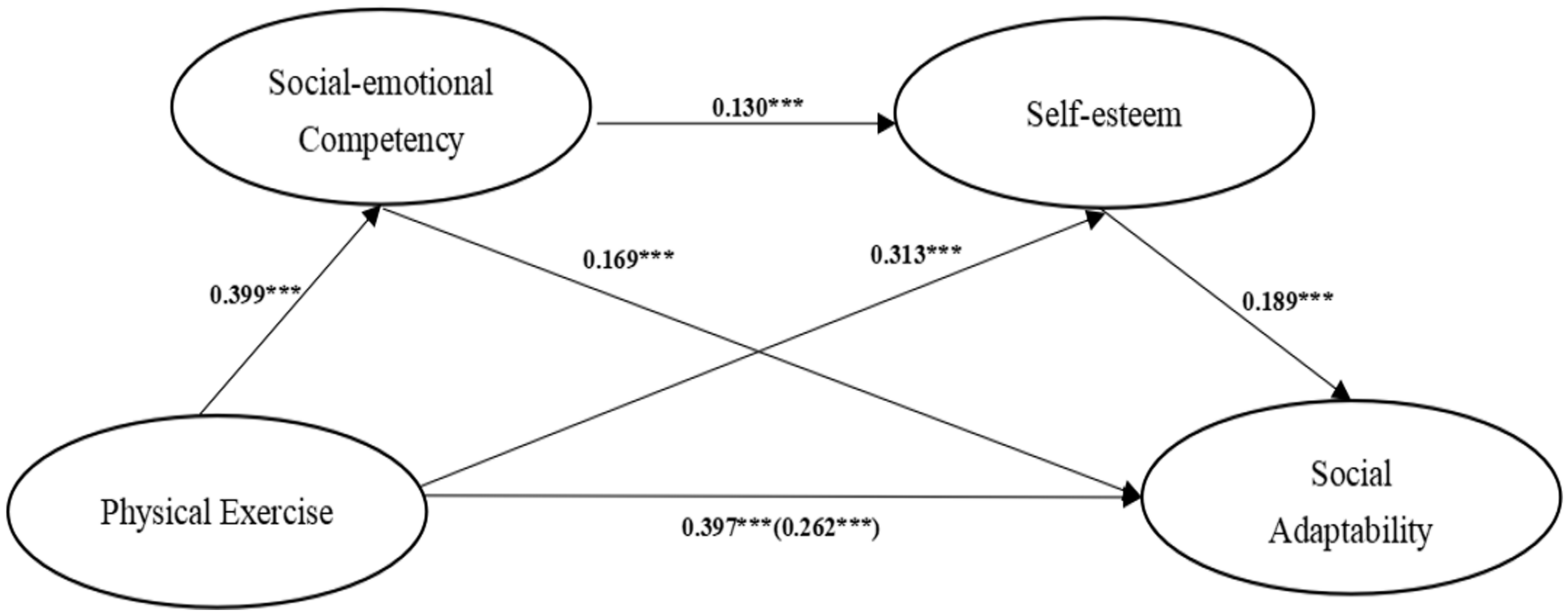
Chain mediation model of social-emotional competency and self-esteem between physical exercise and social adaptability (***p < 0.001).

The deviation-corrected percentile Bootstrap method (repeated sampling 5,000 times) was used to test the mediating effects of social-emotional competency and self-esteem between physical exercise and social adaptability. Results of the mediation effect Bootstrap 95% confidence interval are shown in Table 3. Physical exercise→social-emotional competency→social adaptability, the confidence interval is [0.053, 0.108], excluding 0, and the mediating effect value is 0.068, indicating that social-emotional competency is the mediating variable between physical exercise and social adaptability of college students. Physical exercise→self-esteem→ social-emotional competency, the confidence interval is [0.046, 0.092], excluding 0, and the mediating effect value is 0.059, indicating that self-esteem is the mediating variable between physical exercise and social-emotional competency of college students. Physical exercise→social-emotional competency→self-esteem→social adaptability, the confidence interval is [0.006, 0.018], excluding 0, and the mediating effect value is 0.010, indicating that the chain mediating effect between physical exercise and social adaptability of college students is significant (Figure 2). The mediating effect of social-emotional competency and self-esteem on physical exercise and social adaptability is shown in Figure 2.

**Table 3:** Mediation effect test based on Bootstrap.

		Effect	Boot SE	Boot LLCI	Boot ULCI	Ratio of indirect to total effect
Total effect		0.397	0.032	0.403	0.530	
Direct effect		0.260	0.035	0.240	0.377	65.49%
	Total indirect effect	0.137	0.018	0.125	0.197	34.51%
Indirect effect	Indirect effect 1	0.068	0.014	0.053	0.108	17.13%
	Indirect effect 2	0.059	0.012	0.046	0.092	14.86%
	Indirect effect 3	0.010	0.003	0.006	0.018	2.52%
	Compare 1	0.011	0.020	−0.026	0.052	
	Compare 2	0.068	0.014	0.042	0.097	
	Compare 3	0.056	0.012	0.034	0.081	

Boot SE and Boot LLCI and Boot ULCI, respectively, refer to the standard error of effect and lower and upper limits of 95% confidence interval estimated by Bootstrap deviation correction method. Indirect effect 1: Physical exercise→social-emotional competency→social adaptability; Indirect effect 2: Physical exercise→self-esteem→social adaptability; Indirect effect 3: Physical exercise→social-emotional competency→self-esteem→social adaptability.

## Discussion

This study explores the relationship between physical exercise and college students’ social adaptability, as well as the mediating effect between social-emotional competency and self-esteem. The results show that physical exercise can not only positively affect social adaptability, but also indirectly affect social adaptability through the independent mediating effect of social-emotional competency and self-esteem. Furthermore, physical exercise also affect social adaptability through the chain mediation of social-emotional competency and self-esteem. Besides, this paper further explains the causes of the effect of physical exercise on social adaptability in college students and has certain enlightenment significance for the prevention and intervention of social adaptability in college students.

### The relationship between physical exercise and social adaptability

This study shows that there is a significant positive correlation between physical activity and social adaptability in college students, which is consistent with the results of previous relevant studies (Wu, 2018; Lu and Wang, 2019; Chen and Sto, 2021). Hypothesis 1 is tested. Social adaptability is a kind of ability to realize basic psychological needs, which reflect the individual’s ability to socialize, communicate and do things. Generally speaking, college students who engage in physical exercise actively are better at communicating with others, sharing fun, establishing social interpersonal networks, and developing interpersonal relationships. Physical exercise can not only strengthen the body of college students, but also cultivate their personality and develop interpersonal communication skills. A previous study revealed that physical exercise was the causal variable of adolescents’ social adaptability, while urging and encouraging adolescents to engage in physical exercise activities may effectively improve their social adaptability (Zhu and Shu, 2022).

The experimental researches on the influence of physical exercise on the social adaptability of college students are also consistent with the results of this study. One study found that after the intervention of extracurricular physical exercise, the level of college students’ social adaptability improved significantly, suggesting that extracurricular physical exercise can positively and effectively affect college students’ social adaptability (Lu and Wang, 2019). Studies have also found that the social adaptability of non-physical exercise college students is significantly lower than that of physical training college students. The regression analysis results showed that physical exercise was the most important factor affecting the social adaptability of college students (Wu, 2018). The study by Ji and Zheng (2021) revealed that physical exercise has a positive effect on college students’ mental health and social adaptability. Their study further revealed that the social adaptability of college students is related to the number of times that they participate in sports activities, the duration of a single sports activity, as well as the intensity of sports activities. The more the college students participated in sports activities, the stronger their social adaptability is. Chen and Sto (2021) pointed out that if college students keep the appropriate duration and intensity of sports activities, their interpersonal communication ability and social adaptability will be improved notably. Therefore, physical exercise is beneficial to improve college students’ social adaptability.

### The mediating effect of social-emotional competency

In this study, we find that social-emotional competency plays a mediating role between physical exercise and social adaptability, which verifies hypothesis 2. This finding is consistent with the conclusions of previous studies, namely, physical exercise positively predicts social-emotional competency (Jiang et al., 2018), and social-emotional competency positively predicts social adaptability (Huang et al., 2021). In this study, three variables were examined at the same time, revealing that physical exercise is an important predictor of improving social-emotional competency and social adaptability.

First, this study confirms that physical exercise can positively predict social-emotional competency. Social-emotional competency includes social competency and emotional competency (Choi and Gwag, 2017). Emotional competency is an essential part of social-emotional competency (Beaudoin and Beauchamp, 2020). Physical exercise is an effective way to relieve tension, maintain emotional regulation, help regulate emotional impulses and promote mental health (Wagstaff, 2014). Physical exercise can effectively improve the self-efficacy of emotional regulation, maintain the positive emotions of college students, explore students’ potential, and promote students’ physical and mental health (Yin et al., 2015). Individuals with high self-efficacy are more confident in coping with emotions, especially negative emotions (Caprara et al., 2008). Studies found that emotional regulation self-efficacy also partially mediates the relationship between physical exercise and negative emotions among college students (Tang et al., 2022). Therefore, physical exercise can improve emotional competence through emotional regulation self-efficacy and emotional regulation strategies (Jiang et al., 2018). Poulos and Kulinna (2022) assessed the impact of an after-school curriculum aimed at improving physically active and inclusive play to promote physical, social, and emotional health. They found that, along with physical benefits, engaging in physical activity can support the social and emotional health of youth and promote health and well-being into adulthood.

Secondly, this study shows that social-emotional competency can positively predict social adaptability, that is, the improvement of social-emotional competency will directly promote the enhancement of social adaptability, which is also confirmed by previous studies (Huang et al., 2021). People with higher social-emotional competency often have stronger interpersonal ability, better communication skills with peers, and stronger social adaptability (Allen et al., 2005; Durlak et al., 2011). Domitrovich et al. (2017) pointed out that social-emotional competency is an essential factor for promoting positive adjustment and reducing risk in school children. The study also found that social-emotional competency can significantly predict the social adaptability of middle school students (Huang et al., 2021). People with strong social-emotional competency tend to have higher social ability and emotional regulation ability (Kendziora and Osher, 2016), as well as better psychological health and social adaptability. Therefore, physical exercise can improve the social adaptability of college students by enhancing their social-emotional competency.

### The mediating effect of self-esteem

This study finds that self-esteem plays a mediating role between physical exercise and social adaptability, which verifies hypothesis 3. Previous studies have also confirmed this result, namely, physical exercise positively predicts self-esteem (Ekeland et al., 2004), and self-esteem further positively predicts social adaptability (Huang et al., 2021). In this study, the three variables were investigated at the same time, revealing that physical exercise is not only an important factor to improve individual self-esteem, but also an important factor to improve social adaptability.

This study proves that physical exercise positively predicts the self-esteem of college students. Studies have shown that physical activity positively affects self-esteem (Deng et al., 2022). Regular exercise improves the levels of self-efficacy, self-esteem, and body awareness of young adults (Gulsum et al., 2022). Physical self-concept changed by exercise participation might directly and positively influence mental well-being, and it can indirectly influence the changes in mental well-being *via* improving self-esteem (Kim and Ahn, 2021). Further research have shown that self-esteem was related to physical activity variables, such as physical condition, body mass index (BMI), and level of physical activity (Zurita-Ortega et al., 2017).

This study also confirms that self-esteem is an important predictor of social adaptability. Self-esteem is closely related to social adaptability (Mahadevan et al., 2019). The level of self-esteem of college students has a significant influence on their coping style (Xiong et al., 2008). Individuals with high self-esteem have enough confidence to accomplish something and also have high social adaptability (Zhang, 2013; Li, 2015). A longitudinal study of 642 college students showed that low self-esteem predicted social problems, and low self-esteem uniquely contributes to later social difficulties (Crocker and Luhtanen, 2003). College students with high self-esteem are more positive toward life, more active in face of different environment, more willing to actively adapt to the environment, to meet various challenges, and to achieve higher social adaptability. It is pointed out that positive response support from peers can help individuals achieve the desired goal and improve individual self-esteem (Feeney, 2004). One longitudinal study examined the relationship between self-esteem and depression among Chinese college students during four academic years. The results showed that the self-esteem levels of college students on average monotonically declined over years, and there were significant negative correlations between self-esteem and depression for college students (Gao et al., 2022). Physical exercise is conducive to the establishment of good peer relationships and improves self-esteem. Studies have shown that exercise affects health and also supports a positive mindset of life and self-esteem (Wågan et al., 2021). Moreover, self-esteem enhancement is linked to better social interaction and healthier relationships (He, 2022). Therefore, physical exercise can improve college students’ social adaptability through self-esteem.

### The chain mediation effect of social-emotional competency and self-esteem

In this study, there is a significant positive correlation between social-emotional competency and self-esteem, which is consistent with the research results (Heiman and Olenik-Shemesh, 2020; Huang et al., 2021), further confirming the chain mediating role of social-emotional competency and self-esteem between physical exercise and social adaptability of college students, Hypothesis 4 is verified. It shows that the higher the social-emotional competency of college students is, the higher the level of self-esteem. Social-emotional competency is an important causal variable of self-esteem, which is consistent with previous studies (Huang et al., 2021). On the one hand, college students with higher social-emotional competency are easy to get acceptance and affirmation from the outside. Therefore, their self-concept is more stable and self-esteem is higher. According to the terror management theory put forward by Greenberg et al. (1997), self-esteem can buffer anxiety, and low self-esteem easily lead to individual depression, anxiety, and depression, thus inducing mental illness. Studies reported that people who have higher-quality interpersonal relationships also have higher levels of self-esteem. The overall level of self-esteem of citizens in different countries is positively correlated with the degree of close social interaction characteristics of individuals in the society (Denissen et al., 2008).

On the other hand, cultivating social-emotional competency can improve self-esteem (Heiman and Olenik-Shemesh, 2020), thus improving individual social adaptability. The research shows that social-emotional competency can not only directly improve the social adaptability of middle school students but also indirectly improve the social adaptability of middle school students through peer relationships and self-esteem (Huang et al., 2021). Social-emotional competency can affect social adaptability through self-esteem, maybe because college students with higher social-emotional competency tend to get more recognition in social interactions, and have more stable self-concepts and higher self-esteem. Furthermore, college students with high self-esteem have more positive life attitudes, more active coping ways in the face of problems or difficulties, and higher social adaptability. College students who often take part in physical exercise tend to have higher social-emotional competency and get more recognition in social communication (Wagstaff, 2014; Poulos and Kulinna, 2022). Studies show that people who have higher-quality interpersonal relationships also have higher levels of self-esteem (Denissen et al., 2008). Therefore, physical exercise can improve college students’ social adaptability by improving social-emotional competency and self-esteem. In conclusion, social-emotional competency and self-esteem play a chain mediating role between physical exercise and social adaptability.

### Practical implications

This study examines the relationship between physical exercise and college students’ social adaptability, as well as the mediating role between social-emotional competency and self-esteem. It reveals the influence of physical exercise on college students’ social adaptability and its possible mechanism, which has certain reference significance for improving the mental health of college students. The results of this study suggest that we should not only pay attention to the direct impact of physical exercise on college students’ social adaptability but also improve their self-esteem and further enhance their social adaptability by improving their social-emotional competency through physical exercise. In practice, the following measures can be adopted: First of all, encourage college students to participate in physical activity, provide relevant scientific physical exercise guidance, promote the improvement of college students’ social-emotional competency, self-esteem, and social adaptability, and then further improve the mental health of college students. Secondly, social-emotional competency and self-esteem are essential factors affecting college students’ social adaptability. More attention should pay to the social-emotional competency and self-esteem of college students. We can improve the social-emotional competency and self-esteem of college students by developing some courses of learning social-emotional competency and self-esteem as well as introducing excellent foreign courses.

### Research deficiencies and prospects

The findings of this study have particular theoretical values and practical guidance but also have some shortcomings. First, this study adopted a cross-sectional design because of space and time limitations. The longitudinal follow-up or experimental intervention study can increase in the post-study. Second, there are specific problems with the design and distribution of the questionnaire, making the subjects fill out the questionnaire with some concerns about catering to social acceptance factors. Third, this study only considers the mediating effect of social-emotional competency and self-esteem between physical exercise and social adaptability. Still, there may be other mediating variables that need further research.

## Conclusion

(1) Physical exercise can significantly positively predict college students’ social-emotional competency, self-esteem, and social adaptability, suggesting that physical exercise helps improve college students’ social-emotional competency, self-esteem, and social adaptability. (2) Physical exercise can not only directly affect college students’ social adaptability but also indirectly affect college students’ social adaptability through the independent mediating effect of social-emotional competency and self-esteem. Physical exercise also indirectly affect social adaptability through the chain mediating effect of social-emotional competency and self-esteem. It is suggested that the promotion and intervention of college students’ mental health should not only pay attention to enhancing their attitude and behavior toward physical exercise but also pay attention to improving their social-emotional competency and self-esteem.

## Data availability statement

The original contributions presented in the study are included in the article/supplementary material, further inquiries can be directed to the corresponding author.

## Ethics statement

The studies involving human participants were reviewed and approved by Research Ethics Committee of Zhaoqing University. The patients/participants provided their written informed consent to participate in this study.

## Author contributions

YL and KG designed the study, collected and analyzed the data, and wrote the manuscript. QF and YT revised the manuscript. All authors contributed to the article and approved the submitted version.

## Funding

This research was funded by (1) the Construction and practice of a practical teaching system of Physical Education Specialty in local Normal Colleges under the background of “New Normal” construction (2021GXJK482), Guangdong Province Education Science planning project; (2) the 2021 University-level Scientific Research Fund Project Outstanding young teachers scientific research ability enhancement plan, Research Project of Zhaoqing University; (3) Construction and practice of practical teaching system of Physical Education specialty in local Normal colleges under the background of university transformation (zlgc202135), Teaching Reform project of Zhaoqing University.

## Conflict of interest

The authors declare that the research was conducted in the absence of any commercial or financial relationships that could be construed as a potential conflict of interest.

## Publisher’s note

All claims expressed in this article are solely those of the authors and do not necessarily represent those of their affiliated organizations, or those of the publisher, the editors and the reviewers. Any product that may be evaluated in this article, or claim that may be made by its manufacturer, is not guaranteed or endorsed by the publisher.
